# Workload-dependent hemispheric asymmetries during the emotion-cognition interaction: a close-to-naturalistic fNIRS study

**DOI:** 10.3389/fnrgo.2023.1273810

**Published:** 2023-11-15

**Authors:** Katharina Lingelbach, Sabrina Gado, Maria Wirzberger, Mathias Vukelić

**Affiliations:** ^1^Applied Neurocognitive Systems, Fraunhofer Institute for Industrial Engineering IAO, Stuttgart, Germany; ^2^Applied Neurocognitive Psychology, Carl von Ossietzky University, Oldenburg, Germany; ^3^Experimental Clinical Psychology, Department of Psychology, University of Würzburg, Würzburg, Germany; ^4^Department of Teaching and Learning with Intelligent Systems, University of Stuttgart, Stuttgart, Germany; ^5^LEAD Graduate School and Research Network, University of Tübingen, Tübingen, Germany

**Keywords:** hemispheric asymmetry, lateralization, emotion-cognition interactions, functional near-infrared spectroscopy, workload, emotional speech distraction, inhibitory processes, ecological validity

## Abstract

**Introduction:**

We investigated brain activation patterns of interacting emotional distractions and cognitive processes in a close-to-naturalistic functional near-infrared spectroscopy (fNIRS) study.

**Methods:**

Eighteen participants engaged in a monitoring-control task, mimicking common air traffic controller requirements. The scenario entailed experiencing both low and high workload, while concurrently being exposed to emotional speech distractions of positive, negative, and neutral valence.

**Results:**

Our investigation identified hemispheric asymmetries in prefrontal cortex (PFC) activity during the presentation of negative and positive emotional speech distractions at different workload levels. Thereby, in particular, activation in the left inferior frontal gyrus (IFG) and orbitofrontal cortex (OFC) seems to play a crucial role. Brain activation patterns revealed a cross-over interaction indicating workload-dependent left hemispheric inhibition processes during negative distractions and high workload. For positive emotional distractions under low workload, we observed left-hemispheric PFC recruitment potentially associated with speech-related processes. Furthermore, we found a workload-independent negativity bias for neutral distractions, showing brain activation patterns similar to those of negative distractions.

**Discussion:**

In conclusion, lateralized hemispheric processing, regulating emotional speech distractions and integrating emotional and cognitive processes, is influenced by workload levels and stimulus characteristics. These findings advance our understanding of the factors modulating hemispheric asymmetries during the processing and inhibition of emotional distractions, as well as the interplay between emotion and cognition. Moreover, they emphasize the significance of exploring emotion-cognition interactions in more naturalistic settings to gain a deeper understanding of their implications in real-world application scenarios (e.g., working and learning environments).

## 1 Introduction

Naturalistic environments are typically characterized by a multitude of dynamic visual and auditory stimuli, which are attention-catching signals with biological relevance (Vuilleumier, 2005; Bradley, 2009; Matusz et al., 2019). Previous assumptions about perception, attention, and the underlying brain mechanisms have been derived from traditional research paradigms employing well-controlled, simplified, and isolated experimental stimuli. Thus, these assumptions are often challenged when applied to more naturalistic scenarios, characterized by dynamically changing task demands and interacting cognitive processes (Matusz et al., 2019). Especially, socio-emotional cues like the screaming of children, facial expressions of individuals in close range, or emotional conversations attract attention – regardless of whether they are task-relevant or not – since they reveal salient information for a potential need to adapt one's behavior (Norris et al., 2004; Matusz et al., 2019). Studying the brain patterns underlying such interacting processes in more naturalistic scenarios is decisive, not only for replicating well-controlled laboratory research findings in real-world settings (Peck et al., 2014), but also for the development of safe and efficient human-machine systems (Maiseli et al., 2023).

### 1.1 Effects of emotional distractions on cognitive processes

When a stimulus is salient albeit not relevant to the ongoing task, it competes for cognitive resources with the task-relevant cognitive process and, thereby, potentially decreases task performance (Dolcos et al., 2011; D'Andrea-Penna et al., 2017; Schweizer et al., 2019; Lingelbach et al., 2021). To reduce or even overcome this impairing effect of distractions, coping mechanisms of cognitive control are recruited to re-establish task performance (see Iordan et al., 2013; Schweizer et al., 2019, for reviews). Previous studies have demonstrated that overcoming the disruptive effect of emotionally negative distractions is more challenging compared to non-emotional or emotionally positive distractions (Dolcos and McCarthy, 2006; Dolcos and Denkova, 2014; García-Pacios et al., 2015a,b). This difficulty may arise due to their association with potentially harmful events or threats, making them highly salient. However, the effect of emotionally positive distractions is less investigated and understood (Kellermann et al., 2012; Grimshaw and Carmel, 2014; García-Pacios et al., 2015a,b; Schweizer et al., 2019). Furthermore, previous studies explored the effects of emotionally negative and positive distractions mostly under only one level of workload (e.g., rather low working memory load in García-Pacios et al., 2015a,b). Exceptions investigating emotional distraction effects on several working memory load levels are Erk et al. (2007) and Mano et al. (2013). Using behavioral data, Mano et al. (2013) reported that emotionally positive distractions in the intermediate load level caused the greatest task impairment (but cf., Kellermann et al., 2012). Using both behavioral and neuroimaging data, Erk et al. (2007) studied two levels of working memory load and four distraction conditions: positively, negatively and neutrally rated pictures from the International Affective Picture System (IAPS; Lang et al., 1997), and a control condition (a blank screen). They observed no performance impairment as well as no interference effects on working memory-related brain activation, during emotionally positive and negative distractions. The authors even found improved performance for emotional compared with neutral distractions as well as regulation effects with reduced activity in emotion-processing regions, mediated by prefrontal regions, during high load scenarios (Erk et al., 2007).

### 1.2 Experimental manipulation of emotion and workload

In order to investigate the interaction of different workload levels and emotional distractions, successful manipulations of both factors need to be ensured. Workload is defined as the ratio of available working memory resources relative to the resources required to successfully execute a task (Welford, 1978; Young et al., 2015). The ability to keep and update information in the working memory is limited by both the individual's capacity (Barrouillet et al., 2007) and focus on attention (Kahneman, 1975; Sweller, 1988; Wickens, 2008). To induce certain levels of workload, researchers can adapt the task difficulty level (i.e., information processing demands induced by the task) to increase the amount of cognitive resources required to perform the task. For instance, by increasing the number of information elements that need to be maintained simultaneously in working memory (Baddeley and Hitch, 1974; Sweller, 1988; Wirzberger et al., 2017, 2018). However, this number cannot be increased indefinitely, as this might result in a potential ceiling or overload effect (Cowan, 2001; Mano et al., 2013).

Task-unrelated demands might arise from bottom-up interfering situational aspects, such as the prevalence of distractions competing for an individual's limited processing capacities. To successfully capture a person's attention, a stimulus needs to clearly stand out from the situational background (Wickens et al., 2021). There is evidence that participants' performance in complex cognitive tasks suffers from irrelevant but intelligible speech stimuli (Banbury and Berry, 1998; Liebl et al., 2012). Human speech is a natural and essential means of communication, conveying not only linguistic information but also reflecting the speaker's emotional state (Chen et al., 2016). Importantly, strong emotional intensity with associated acoustic characteristics contributes to stimulus salience (Anikin, 2020) and one may expect that this exacerbates the disruptive effect. However, in their meta-analysis, Schweizer et al. (2019) highlighted that a behavioral effect of emotional distraction is difficult to capture due to coping mechanisms counteracting task-related impairments, particularly in healthy samples.

Even when coping efforts veil behavioral effects, these may still be observable in brain activation patterns, which may capture interacting processes involved in the perception of emotional distractions and maintenance of goal-directed behavior (e.g., Babiloni, 2019).

### 1.3 Theories and neurophysiological correlates of emotional and cognitive processes

When investigating neuronal activation patterns associated with workload and emotional distraction, the prefrontal cortex (PFC) is a key region for goal-directed behavior and executive functions, such as information retrieval and updating, action monitoring, and inhibition (e.g., Miller et al., 2002; Kondo et al., 2004; Erk et al., 2007; Asplund et al., 2010; Dehais et al., 2020). The fronto-parietal control network, a functional connection between frontal and parietal regions, is recruited during task-related working memory maintenance (Curtis, 2006; Martínez-Vázquez and Gail, 2018). Thereby, the involvement of the right dorsolateral prefrontal cortex (dlPFC) is especially crucial (Pessoa et al., 2002; Curtis and D'Esposito, 2003; Nee et al., 2007; Barbey et al., 2013; Schweizer et al., 2019). The ventrolateral prefrontal (vlPFC) and orbitofrontal cortex (OFC) are recruited during the inhibition of emotional distractions and their activation is correlated with task performance (Ochsner et al., 2004; Iordan et al., 2013; Eden et al., 2015).

For stimuli such as words or pictures, which were identified to be meaningful, the left inferior frontal gyrus (IFG) is proposed to be involved in the encoding and classification of semantic categories (Gabrieli et al., 1998; Zahn et al., 2000; Wildgruber et al., 2004; Friederici, 2012). Additionally, during the emotional valence processing of semantic stimuli, greater activation was observed in the left hemisphere when presented with positive compared to negative stimuli (Ahern and Schwartz, 1985; Silberman and Weingartner, 1986; Harmon-Jones and Allen, 1998; Herrington et al., 2005; Smith et al., 2017). This so-called *valence theory*, proposing an emotion-specific frontal hemispheric asymmetry (e.g., Berntson et al., 2011; Grimshaw and Carmel, 2014; Güntürkün et al., 2020) mainly stems from research using electroencephalography (EEG; Davidson, 1992, 1993; Smith et al., 2017). However, it has also been partially observed in studies utilizing functional magnetic resonance imaging (fMRI; Canli et al., 1998; Beraha et al., 2012) and functional near-infrared spectroscopy (fNIRS; Balconi and Vanutelli, 2016; Balconi et al., 2017; Hu et al., 2019).

Hemispheric asymmetries, encompassing both structural and functional differences, constitute a fundamental principle in cerebral organization, reflecting the relative dominance of one hemisphere over the other in a cognitive process (Toga and Thompson, 2003; Hugdahl, 2011; Ocklenburg et al., 2016; Esteves et al., 2020; Güntürkün et al., 2020). A prominent example of hemispheric dominance is observed in hand motor control: right-handed individuals exhibit left-lateralized processing, while in left-handed individuals, hemispheric dominance can vary (Ziemann and Hallett, 2001; Ocklenburg et al., 2013; Guadalupe et al., 2014; McManus, 2019). Language is another notable instance of hemispheric asymmetry, predominantly displaying left-hemispheric dominance (Corballis, 2012; Greve et al., 2013; Ocklenburg et al., 2014; Wang et al., 2018). Nevertheless, the right hemisphere also plays a vital role in processing specific linguistic aspects, such as prosody (Lindell, 2006; Güntürkün et al., 2020). Additionally, right hemispheric dominance is observed in various other functions, including face and body perception (Meng et al., 2012; Dundas et al., 2013; Thoma et al., 2014) as well as (visual-)spatial attention and processing (Corbetta and Shulman, 2011; Thiebaut de Schotten et al., 2011; Chen and Spence, 2017; Bartolomeo and Seidel Malkinson, 2019).

In order to investigate hemispheric asymmetries, fNIRS provides a suitable, non-invasive optical technology that measures concentration changes of local oxygenated hemoglobin (HbO) and deoxygenated hemoglobin (HbR) caused by the current metabolic demand of the cortical brain region (Ferrari and Quaresima, 2012; Pinti et al., 2020). With its relatively high spatial resolution and usability, which also facilitates mobile data acquisition, it is gaining growing attention as a means to investigate the hemodynamic brain response under more naturalistic conditions (Ayaz et al., 2012; Benerradi et al., 2019; von Lühmann et al., 2020, 2021).

#### 1.3.1 The asymmetric inhibition model for emotional distractions

It is essential to highlight that most theories of emotion processing, such as the valence theory (e.g., Herrington et al., 2005; Berntson et al., 2011; Smith et al., 2017) or motivational direction theory (e.g., Sutton and Davidson, 1997; Harmon-Jones and Allen, 1998), do not explicitly incorporate the interaction of emotional and cognitive processes. Instead, they tend to characterize emotional processing in isolation, neglecting to examine the interplay between cognitive control mechanisms and emotional processes. For example, results inconsistent with these established theories of emotion processing have been found in studies investigating the regulation of emotions (e.g., Compton et al., 2003; Pérez-Edgar et al., 2013).

An attempt to explain goal-directed behavior with successful task maintenance and the underlying brain processes during emotional distractions is made by the *asymmetric inhibition model* (Grimshaw and Carmel, 2014). The model posits asymmetries in executive control mechanisms, where right-lateralized executive control inhibits the processing of positive or approach-related distractions, while left-lateralized control serves to inhibit negative or withdrawal-related distractions. The relationship between the dominant hemisphere and valence is proposed to be reversed when it comes to inhibiting task-irrelevant emotions, in contrast to models that explain the processing of these emotions (Grimshaw and Carmel, 2014). The authors emphasize the role of the lateral PFC during the inhibition of task-irrelevant emotions (Gray et al., 2002; Wager et al., 2003; Ochsner et al., 2012; Grimshaw and Carmel, 2014). Their assumptions are mainly derived from EEG and fMRI research with healthy and clinical study samples showing the reversed asymmetric hemispheric processing (Compton et al., 2003; Pérez-Edgar et al., 2013).

Gray et al. (2002) investigated the integration of cognitive processes and emotions and described the process as a convergence where specialized sub-functions merge into a single, more general function. Their findings are in line with the asymmetric inhibition model (Grimshaw and Carmel, 2014), showing greater right lateral PFC activation during pleasant emotions and left-hemisphere activation during unpleasant emotions. Importantly, the authors emphasize that a crossover interaction without significant main effects for workload or emotion is required to assume the integration of cognitive processes and emotions. This indicates that the observed neuronal activity is not predictable solely from information about either factor (workload or emotion) on its own. Instead, it is influenced by the combined interaction of these two factors (Gray et al., 2002).

At the current state, we have identified three aspects of the asymmetric inhibition model that warrant further investigation: First, the model, along with its supporting evidence, lacks specificity concerning the integration of emotion and cognition at different levels of workload. Second, the model's assumptions have yet to be examined in any study employing fNIRS. Third, it does not fully account for stimuli, such as concurrent emotional speech distractions, that may trigger implicit lateralized processing in addition to the inhibitory cognitive control processes.

### 1.4 Research question and hypotheses

Despite first insights into integrating emotions and cognitive processes, evidence remains inconclusive, especially concerning the impact of increasing workload levels and naturalistic speech-related (socio-)emotional distractions on hemispheric asymmetries of PFC activity.

In this study, our aim was to investigate the neurophysiological correlates that capture interactions between emotional and cognitive processes in a naturalistic experimental scenario using fNIRS. More precisely, we examined the effects of concurrent emotional speech distractions (positive, negative, or neutral) on cognitive processes under low and high workload.

We compared the observed brain activation patterns with the assumptions made by the asymmetric inhibition model (Grimshaw and Carmel, 2014). We investigated whether the model assumptions hold true for (a) naturalistic auditory emotional speech distractions presented concurrently with the task and (b) increasing workload levels potentially triggering a re-evaluation of distraction toward a more negative perception.

Our hypothesis was that the integration of emotion and cognitive process manifests in a significant interaction of valence of the auditory distractions and workload level (Gray et al., 2002; Grimshaw and Carmel, 2014).

1. *Hypothesis*: Based on Grimshaw and Carmel (2014), emotionally negative distractions should elicit stronger activation in the left lateral PFC compared to positive distractions; the activation pattern is expected to remain independent of the concurrent workload level.
2. *Hypothesis*: Emotionally positive distractions are assumed to elicit stronger activation in the right lateral PFC compared to negative distractions; the activation pattern is expected to remain independent of the concurrent workload level.
3. *Hypothesis*: Emotionally neutral distractions are expected to result in reduced activation in the left lateral PFC compared to negative distractions and reduced activation in the right lateral PFC compared to positive distractions in the low workload condition. However, based on observations in a feasibility study with EEG (Lingelbach et al., 2021), we hypothesize a *workload-dependent effect* for task-irrelevant neutral distractions. Under high workload, neutral distractions are assumed to be evaluated as rather emotionally negative, thereby triggering similar neuronal activation patterns to emotionally negative distractions, with stronger activation in the left lateral PFC compared with positive distractions.

- *Exploratory hypothesis*: Given that naturalistic emotional speech distractions have been shown to trigger semantic processing and evaluation associated with left-hemispheric activation in the IFG (Wildgruber et al., 2004; Friederici, 2012), it is possible that we observe a bias toward left lateral PFC activation (*emotional speech-dependent effect*). However, a potential prerequisite for this phenomenon could be the availability of sufficient cognitive resources to comprehensively process the emotional speech distractions. Therefore, we explore whether we observe a bias toward left lateral PFC activation for positive distractions during low workload conditions. Such a finding would contradict the assumptions of the asymmetric inhibition model, highlighting potential stimulus-specific dependencies.

## 2 Methods

### 2.1 Participants

Volunteers were recruited via emails through mailing lists as well as social media platforms and screened for eligibility. Persons reporting insufficient German language skills, limited color vision, pronounced alcohol consumption, mental, neurological, or cardiovascular diseases as well as pregnancy were excluded. Twenty participants took part in the study. Due to minor changes in the experimental procedure, we had to exclude the first two participants, leaving a final sample of nine female and nine male participants (mean age *M* = 25.9 years, *SD* = 3.8, range = 21–35; 15 right-handed and 3 left-handed). All participants had a normal or corrected-to-normal vision, received financial compensation for their participation, and signed an informed consent in accordance with the recommendations of the Declaration of Helsinki.

### 2.2 Procedure

Participants first familiarized themselves with the experimental task in a practice run. In the experiment, they had to perform monitoring-control tasks of an air traffic controller while their brain activity was recorded via fNIRS.

#### 2.2.1 Neurophysiological measures via fNIRS

**Figure 1:**
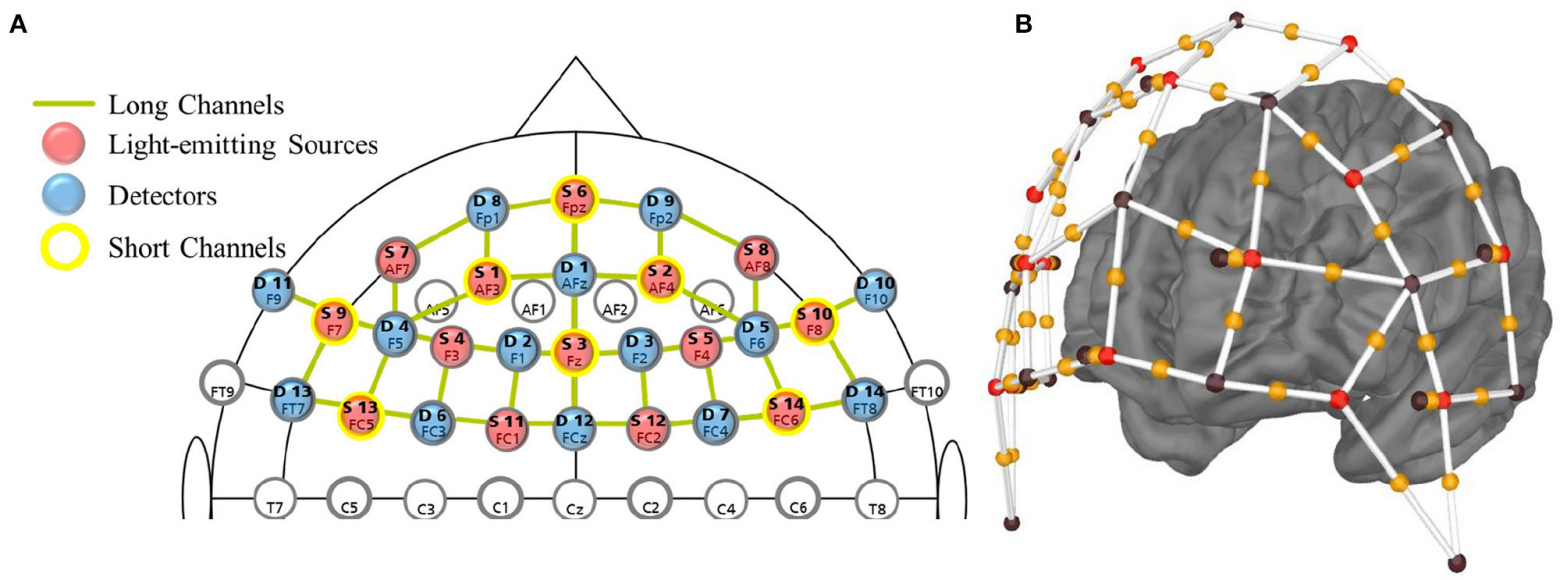
**(A)** Montage of optodes on the fNIRS cap using the standard 10–20 system for sensor location. Labels include the source or detector number (bold) and 10–20 system position. **(B)** 3D view of the montage. Red spheres: near-infrared light-emitting sources; brown spheres: detectors; orange spheres: long channels; red-orange-brown sphere pairs: short channels.

Data were recorded using the NIRx NIRSport2 system (dual LEDs emitting light at two wavelengths: 760 and 850 nm) with the Aurora fNIRS recording software at a sampling rate of 5.8 Hz. The montage with 14 sources and 14 detectors was determined using the fNIRS Optodes' Location Decider (fOLD) toolbox (Zimeo Morais et al., 2018) in order to optimally investigate the brain regions of interest i.e., the OFC [Brodmann Area (BA) 11], dlPFC [BA 9, 46] and vlPFC, comprised of the IFG [BA 47] and Broca's Area [BA 44, 45]; as localized by fOLD. The setup comprised 41 source-detector long channels (hereafter named channels) and eight short channels (Figure 1). An overview of the channels, MNI coordinates, and Brodmann Area correspondences is provided in the Supplementary Table 1.

#### 2.2.2 Experimental task

**Figure 2:**
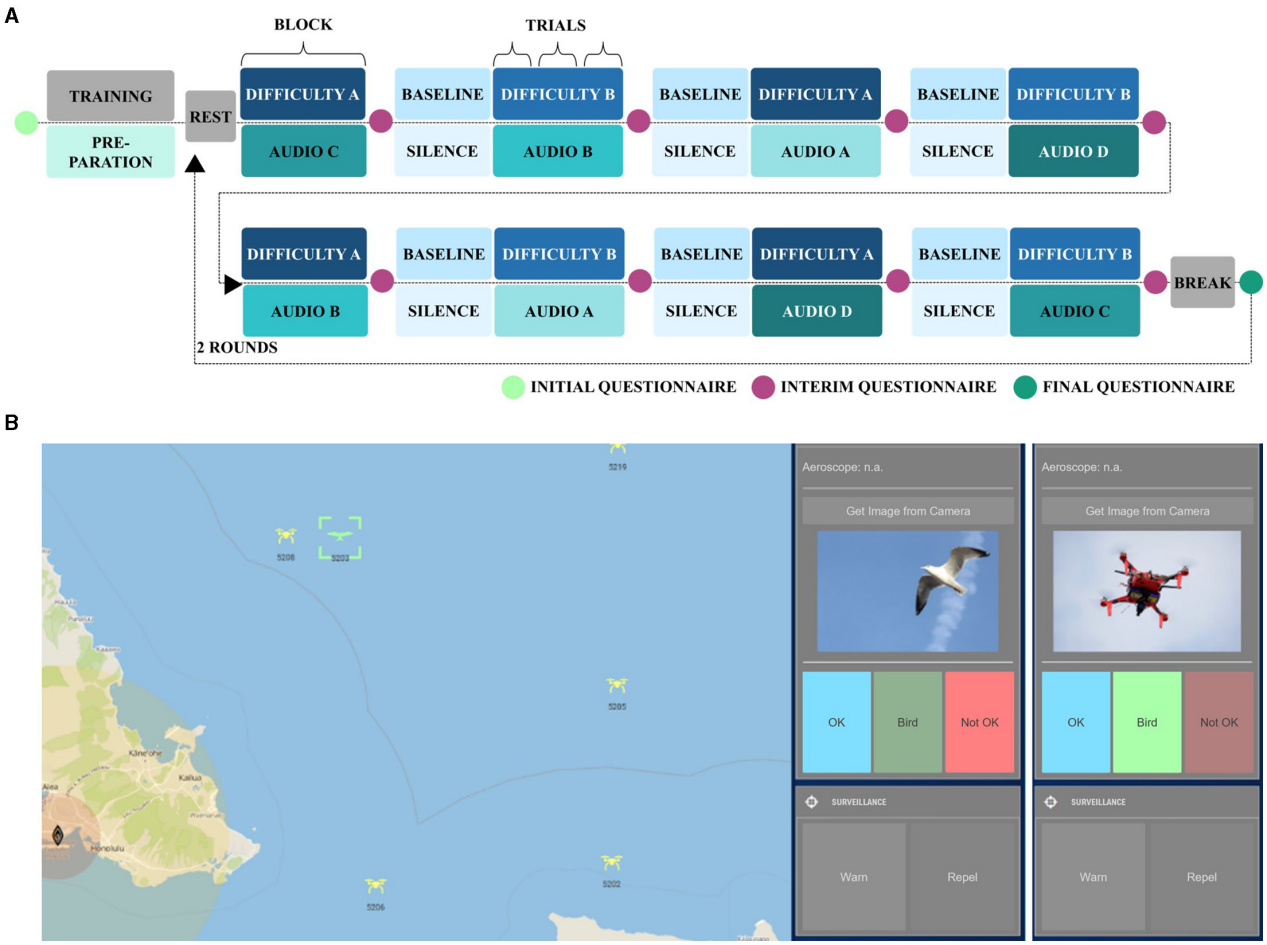
**(A)** Procedure of the block-design experiment with two difficulties (A and B, i.e., low and high workload) and four auditory distractions (A, B, C, and D, i.e., negative, neutral, positive, and silence). The presented procedure is illustrative as the workload was alternating and concurrent emotional distraction was randomized in each round. **(B)** Illustrative screenshot of the graphical user interface of the experimental task. Users initially had to perform an identification query for each uncategorized object on the interactive radar screen (left) using “Aeroscope” in the control panel (right). This step allowed registered drones to be distinguished from unregistered objects. In cases where no registration code was available, users were prompted to request a camera image. If the camera image showed a bird, the object was to be categorized as “bird” (left). If the camera image revealed a drone, it was necessary to assign the label “Not OK” (right) to identify it as a potentially threatening drone. If an unregistered safety-critical drone entered the highlighted radius in close proximity to the airport, users were required to respond accordingly by issuing a warning (yellow circle) and taking measures to repel the drone (red circle).

The experimental task was an adapted version of the warship commander task (WCT, John et al., 2002; adapted by Becker et al., 2021), which is a quasi-realistic navy command and control task. In our version, we used a non-military and safety-critical context. Participants monitored a simulated radar screen close to an airport and performed the following subtasks for each uncategorized object displayed on the radar screen (yellow objects in Figure 2B): (1) object detection, (2) object categorization, (3) rule application and decision-making, and (4) rule-based action (Becker et al., 2021). Objects were categorized as either neutral (i.e., birds; green in Figure 2B), registered drones (blue in Figure 2B), or non-registered, thus, safety-critical drones (red in Figure 2B). For the latter, when entering certain ranges close to the airport, participants had to first warn (yellow circle) and, then, repel (red circle) them (see Becker et al., 2021, for a more detailed description). Concurrently during the task, we presented auditory emotional distractions. For these, we concatenated either emotionally positive, negative, or neutral spoken vocal utterances of 2 s length from the validated Berlin Database of Emotional Speech (Emo-DB; Burkhardt et al., 2005) into 1-min audio sequences of each emotional condition. The stimuli of the Emo-DB can be accessed online at https://audeering.github.io/datasets/datasets/emodb.html. The database consists of ten neutral phrases extracted from everyday conversations, each spoken by ten different actors with varying emotional tones, including anger, neutrality, and joy. Examples of these phrases are: “What about the bags standing there under the table?” (English translation; German original: “Was sind denn das für Tüten, die da unter dem Tisch stehen?”) and “The black sheet of paper is located up there besides the piece of timber.” (“Das schwarze Stück Papier befindet sich da oben neben dem Holzstück.”; Burkhardt et al., 2005). Thereby, speakers and phrases of the 2 s sub-sequences were randomly selected with as little repetition as possible within each of the 1-min audio sequences. We used a 2 (low and high) × 4 (negative, neutral, positive, and silence) within-subject block design with eight experimental conditions. Workload was manipulated via task difficulty by implementing two difficulty levels with 12 to-be-tracked objects (six non-registered drones) in the low workload and 36 to-be-tracked objects (17 non-registered drones) in the high workload condition. Participants completed two rounds, each comprising eight blocks, with three 1-min trials of the same experimental condition (Figure 2A). Workload conditions alternated across blocks, with the start condition alternating across participants. The order of the emotional distraction conditions was randomized across blocks with no repetition of the same condition within a round. Each round started with a resting state measurement of 30 s. Before each block, except for the first, participants completed an active baseline condition of very low workload with six objects (three non-registered drones) and no emotional distraction (silence). Participants' performance was quantified using their reaction time as well as the accuracy of their actions. The accuracy was operationalized trial-wise with a score ranging from 0 (low accuracy–low performance) to 1 (high accuracy–high performance). The score was computed based on the number of hits, correct rejections, misses, and false alarms, divided by the maximum achievable score (Becker et al., 2021). Similarly, the score of the reaction time was computed as a ratio of achieved points and the maximum of possible points ranging from 0 (high reaction time–low performance) to 1 (low reaction time–high performance). Participants received credit points for the timely repelling of a non-registered drone (<10 s), but also obtained penalty points for missed or delayed necessary actions (Becker et al., 2021). After each experimental block, participants rated their subjectively perceived effort (NASA TLX subscale ranging from 0 to 20; adapted from Hart and Staveland, 1988), arousal and valence (EmojiGrid ranging from 0 to 10; Toet et al., 2018).

### 2.3 Statistical analysis

#### 2.3.1 fNIRS preprocessing

The fNIRS signals were preprocessed using the MNE-Python (1.1.1; Gramfort et al., 2014) and MNE-NIRS (0.2.1; Luke et al., 2021) toolboxes, following guidelines by Yücel et al. (2021). Only experimental conditions with auditory distraction were included in the following analyses. Additional analyses investigating task-induced and distraction-induced workload by including the silence condition can be found in Supplementary Figure S4.

In the first step, raw data were converted into an optical density measure. We applied channel pruning using the scalp-coupling-index as a quality measure (Pollonini et al., 2014), with a threshold below 0.5 to identify and exclude channels with poor quality. Next, we performed a temporal derivative distribution repair to account for baseline shifts and spike artifacts (Fishburn et al., 2019). Afterwards, the modified Beer-Lambert Law was used to transform the optical density data into HbO and HbR concentration changes with a partial pathlength factor of 6 (Huppert et al., 2009a; Gramfort et al., 2014; Luke et al., 2021). Data were then filtered using a fourth-order zero-phase Butterworth bandpass filter with cutoff frequencies of 0.05 and 0.7 Hz and a transition bandwidth of 0.02 and 0.2 Hz to remove instrumental and physiological noise (such as heartbeat and respiration). In the last preprocessing step, we applied a negative correlation enhancement algorithm (Luke et al., 2021) to further improve the NIRS signal based on the principle that the true HbO and HbR signals should be negatively correlated (Cui et al., 2010).

#### 2.3.2 First-level generalized linear models

To investigate the effects of emotional distractions and workload levels, we used first-level generalized linear models (GLM) to model the hemodynamic response during the experimental conditions for each participant using the 60 s long trials and a canonical statistical parametric map hemodynamic response function (HRF; Luke et al., 2021; von Lühmann et al., 2021; Yücel et al., 2021). The GLM approach allows extracting estimates for each experimental condition and channel. To correct for systemic signals contaminating the brain activity measured in the long channels, the HbO and HbR short channel signals were added as regressors in the first-level GLM (Saager and Berger, 2005; Santosa et al., 2020; Yücel et al., 2021). In our analysis, we further added a third-order polynomial drift as a regressor to model low-frequency oscillations in the signals (Yücel et al., 2021) as well as the active baseline and resting state to account for interindividual variability. An illustrative example of the GLM design matrix is provided in the Supplementary Figure S1.

Estimates for the contrasts of interest were obtained participant-wise using the GLM coefficients (Abraham et al., 2014).

#### 2.3.3 Second-level linear mixed-effects models

The fNIRS second-level analysis was performed with *R* (version 4.1.1) and python^*TM*^ (version 3.7.7). We used linear mixed-effects models (LMM; Baayen et al., 2008) to estimate second-level coefficients based on the participant-wise *z*-standardized first-level GLM coefficients using the *R* packages *lme4* (version 1.1-27.1; Bates et al., 2015) and *lmerTest* (version 3.1-3, Kuznetsova et al., 2017). We included participants as random intercepts in the models to account for non-systematic interindividual differences. The LMMs provided a second-level estimate per channel for each chromophore (HbO and HbR) and contrast (Luke et al., 2021). We first tested contrasts determining interaction effects, and if the interaction was non-significant, we proceeded to examine the main emotion and workload effects by averaging over one factor, respectively. To determine significant channels, we performed bootstrapping with 5,000 iterations to calculate the 2.5*th* and 97.5*th* confidence interval (CI) of the estimates (Cumming and Finch, 2005). The CI was Bonferroni–corrected for multiple comparisons using the channel number. For a significant effect, the CI must not include zero (Cumming and Finch, 2005). Significant standardized second-level coefficients were visualized by projecting them onto a 3-dimensional average brain template from both the rostral and lateral perspectives (Gramfort et al., 2014). To interpret the interaction effects, we further calculated and visualized (a) the contrasts of the respective subconditions involved in the interaction (hereafter referred to as subcontrasts) as well as (b) the bootstrapped grand average of the first-level GLM estimates, along with its CI across participants per subcondition for significant channels (Cumming and Finch, 2005; Garofalo et al., 2022).

#### 2.3.4 Behavioral data

The behavioral data include the subjective ratings of effort, arousal, and valence, as well as performance measures, specifically reaction time and accuracy. We analyzed the interaction or main effects between emotional distractions and workload using non-parametric bootstrapping and calculated grand averages and their confidence intervals of the contrasts (Cumming and Finch, 2005). We calculated the contrasts of interest per participant, starting with interaction effects. We again proceeded to examine the behavioral main effects of emotional distraction and workload only in cases where the interaction was non-significant. The grand average across participants and the mean's CI per contrast was estimated using bootstrapping with 5,000 iterations. Contrasts with their mean's CI not including zero are considered significant (Cumming and Finch, 2005).

## 3 Results

An overview of all fNIRS interaction or main effects along with their subcontrasts can be found in Supplementary Figure S2 for the HbO and Supplementary Figure S3 for the HbR estimates. To assess the potential impact of handedness on lateralized brain activity in the observed effects (Ziemann and Hallett, 2001; Ocklenburg et al., 2013; Guadalupe et al., 2014; O'Regan and Serrien, 2018; McManus, 2019), we reanalyzed the second-level LMMs, incorporating handedness as an additional fixed factor. The results indicate that handedness had no significant effect in any of the analyses (Supplementary Table 2).

### 3.1 Interaction effects of emotional distractions and workload

There were no significant interaction effects in the HbO estimates (Supplementary Figure S2) or behavioral data (Supplementary Figures S5, S6).

**Figure 3:**
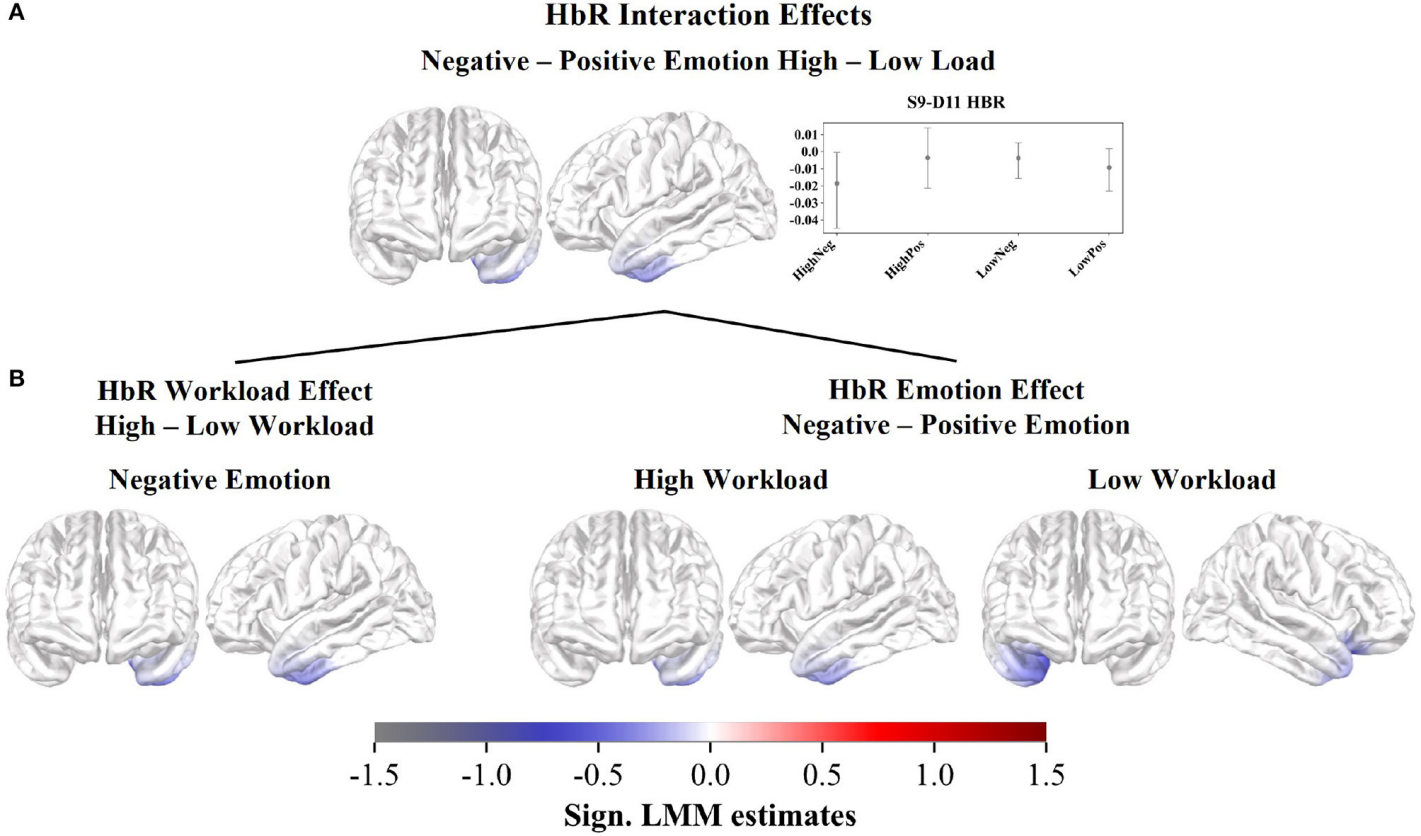
Cortical rostral and lateral surface projections of the significant standardized LMM HbR estimates in the interaction effect. **(A)** Bootstrapped mean first-level GLM estimates and confidence intervals (CIs) across participants per subcondition within the significant channel S9-D11. **(B)** Subcontrasts of the interactions calculated between the subconditions and devided in workload and emotion-related effects.

However, we observed a significant but small interaction effect in the HbR estimates for negative against positive distractions and workload levels (Figure 3; Supplementary Table 3). The interaction revealed a cross-over effect with activity changes in the left IFG and left temporopolar regions. When comparing the average mean first-level GLM estimate and its confidence interval in the significant channel per subcondition (Figure 3A), the interaction is characterized by lower estimates for negative distractions during high workload and positive distractions during low workload. Whereas, higher estimates are observed for positive distractions during high workload and negative distractions during low workload. The significant subcontrasts of the interaction further illustrate decreased HbR estimates, hence increased brain activity, in the left IFG, during negative distractions with high workload, compared to both negative distractions during low workload and positive distractions during high workload (Figure 3B).

### 3.2 Main effects of emotional distractions

Since we did not find any interaction effects in the HbO estimates or for the HbR contrasts between neutral and negative, as well as neutral and positive distractions, we proceeded to test for these main effects of emotional distraction.

**Figure 4:**
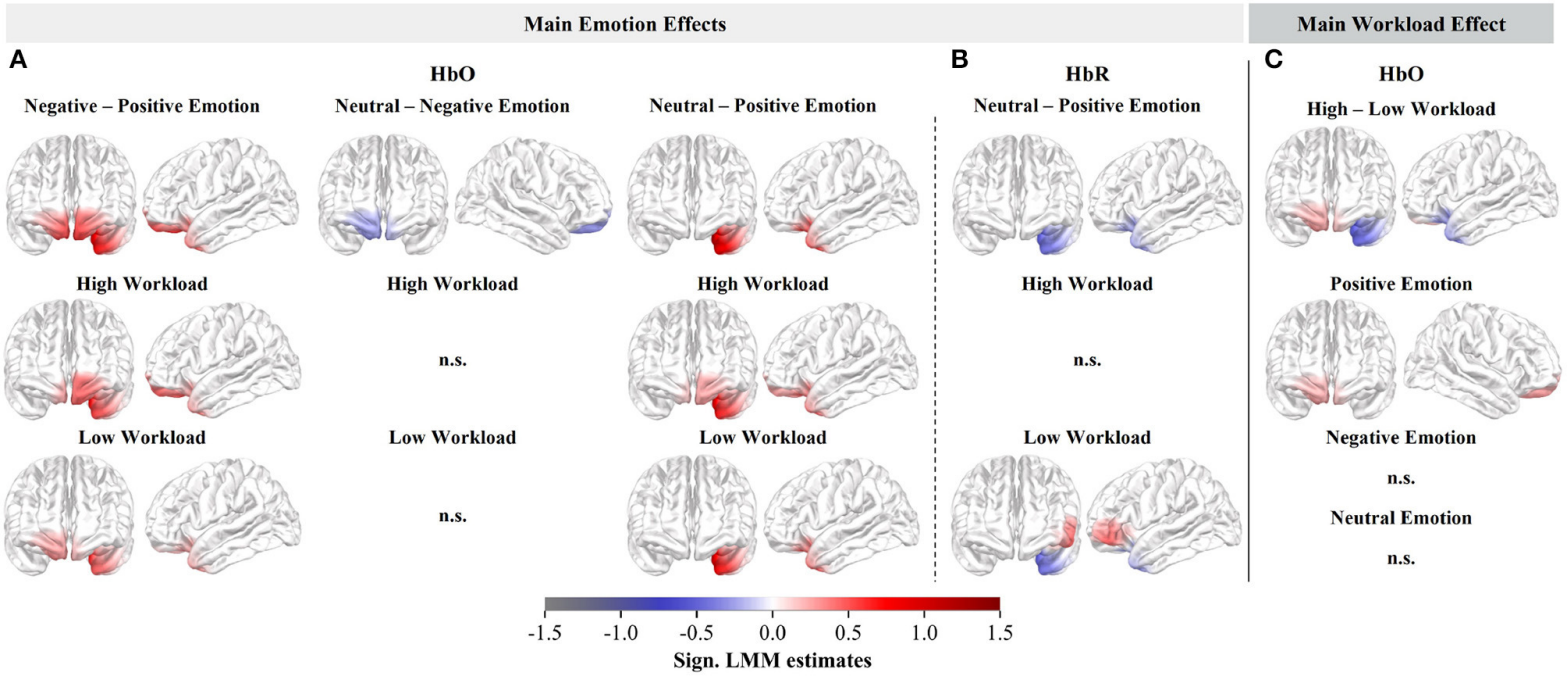
Cortical rostral and lateral surface projections of the standardized second-level LMM estimates. Significant HbO **(A)** and HbR **(B)** estimates of the main emotion effects (averaged across workload) as well as the respective subcontrasts (per workload level). **(C)** Significant HbO estimates of the main workload effect (averaged across emotions) and its respective subcontrasts (per emotion).

We observed significant differences in the HbO estimates for all three main emotion contrasts and in the HbR estimates for neutral compared to positive emotional distractions (Figures 4A, B, upper row). The HbO comparisons between negative and positive distractions, as well as neutral and positive distractions, revealed similar activation patterns, exhibiting significantly higher HbO estimates and stronger recruitment of the left OFC during negative and neutral distractions (Figure 4A, upper row). Consistent with the HbO results, lower HbR in the left OFC was observed during neutral compared to positive distractions (Figure 4B, upper row). Additionally, comparing negative and positive distractions showed bilateral frontopolar HbO increases (Figure 4A, upper row, left). Lastly, a small effect in the HbO responses with decreased frontopolar involvement was observed when comparing neutral and negative distractions. However, this effect vanished when analyzing the subcontrasts per workload level (Figure 4A, middle).

#### 3.2.1 Behavioral effects of emotional distractions

**Figure 5:**
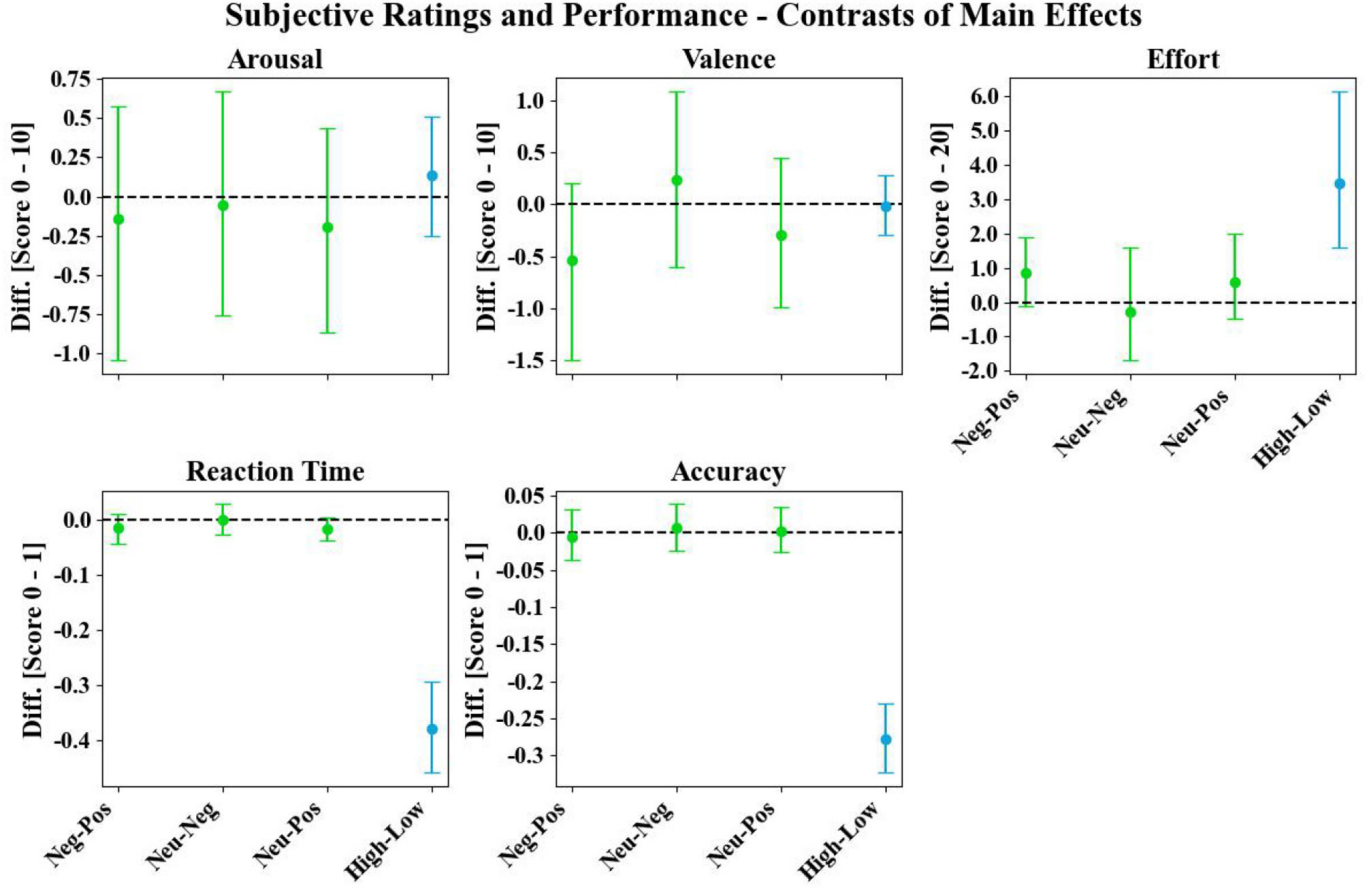
Main emotion (green error bars) and workload (blue error bars) contrasts of the subjective arousal and valence ratings (scale range: 0–10), subjective effort (scale range: 0 to 20), reaction time (scale range: 0–1), and accuracy (scale range: 0–1). Dots and error bars represent the bootstrapped grand averages and their Bonferroni-corrected 2.5th and 97.5th confidence interval (CI) across participants.

When examining the behavioral data, we observed two non-significant trends of main emotion effects: First, negative distractions led to an increase in subjective effort compared to positive distractions. Second, a decrease in performance with longer reaction times was observed for neutral and negative distractions compared to positive distractions (green error bars; Figure 5).

### 3.3 Main effects of workload

Due to the significant interaction effect between emotional distractions and workload in the HbR responses, we solely focused on testing the main workload effect in the HbO. We observed a significant HbO main effect of workload, with a slight increase of HbO, hence increased activity, in the right frontopolar cortex, and a decrease in the left OFC, during high as opposed to low workload (Figure 4C, upper row).

#### 3.3.1 Behavioral effects of workload

In the behavioral data, a robust modulation was observed for all workload-related measures, with increased subjective effort and decreased performance (reaction time and accuracy) during high compared to low workload (blue error bars; Figure 5).

## 4 Discussion

Our study investigates interacting cognitive and emotional processes by examining the brain activation patterns of emotional auditory distractions (positive, negative, and neutral) on cognitive processes under low and high workload. We opted for a close-to-naturalistic experimental design, which involved a monitoring-control task simulating typical air traffic controller requirements, while emotional speech served as distractions.

### 4.1 Workload-dependent hemispheric asymmetries for negative distractions

We hypothesized that the integration of processing emotional distractions and workload levels should manifest in a significant interaction of the two factors (Gray et al., 2002). Our results revealed a significant but small interaction effect in the HbR concentration changes. Similar effect patterns could not be observed in the HbO concentration changes.

The HbR interaction indicates the involvement of the left IFG during the integration of negative distractions compared to positive distractions and the two workload levels. The workload level significantly modulated the left IFG recruitment.

We expected stronger left-hemispheric recruitment during negative compared to positive distractions (Hypothesis 1). However, when examining the HbR estimates, the activation pattern interacted with the workload level. Negative distractions triggered left-hemispheric processing only during high workload scenarios, and left-hemispheric processing even decreased for negative distractions compared to positive distractions in low workload scenarios. Interestingly, the HbR response of the subcontrast between negative and positive distractions during low workload indicated an increased involvement of the right IFG and OFC during negative distractions. From these observations, one might conclude that a certain level of workload is necessary to trigger left-hemispheric inhibitory processes during negative distractions, consistent with the asymmetric inhibition model. During lower workload levels, when sufficient processing capacities are available, the auditory emotional stimulation might not have been perceived as a distraction requiring inhibition. In such a scenario, emotional stimuli might have been processed according to the valence theory (Davidson, 1993; Berntson et al., 2011; Smith et al., 2017), triggering right hemispheric processing during negative stimuli and left hemispheric processing during positive ones. However, inconsistencies between the HbR and HbO effects challenge the interpretation of the results. Although we did not observe an HbO interaction effect, the activation patterns of the main emotion effect support left-hemispheric processing during negative distractions, in agreement with the asymmetric inhibition model (Grimshaw and Carmel, 2014).

Apart from activation patterns in the IFG and OFC, negative distractions evoked bilateral frontopolar activity when compared to positive distractions and right frontopolar activity when compared to neutral distractions (HbO main emotion effects). The frontopolar cortex is suggested to play a crucial role in goal-oriented executive functions and cognitive control (Niendam et al., 2012), particularly in prioritizing competing goals and multiple subtasks (Mansouri et al., 2017). Anticevic et al. (2010) reported a connection between performance and reduced deactivation in the frontopolar cortex, suggesting its involvement in resisting negative interference during cognitive tasks. Similarly, Feng et al. (2021) found decreased frontopolar activation and performance during negative compared to neutral affective states in higher working memory load levels, indicating a failure to resist the negative interference. Consequently, the frontopolar activation observed in our study during negative distractions compared to positive and neutral distractions might indicate the engagement of cognitive control processes. These frontopolar processes could potentially work in tandem with emotion-specific hemispheric inhibitory processes and aim at prioritizing the workload task while effectively overcoming task-irrelevant distractions.

To conclude, we observed the expected left hemispheric processing with involvement of the left OFC and IFG, as well as frontopolar areas, during negative distractions. However, left hemispheric processing was particularly prominent during high workload, suggesting that increased workload enhances the asymmetric processing effect elicited by negative distractions.

### 4.2 Right-hemispheric processing for positive distractions

We did not observe clear right-hemispheric recruitment during positive compared to negative distractions and have to reject our second hypothesis. We even observed decreased right OFC and IFG activity in the HbR subcontrast of low workload when comparing negative and positive distractions. However, when investigating the HbO main workload effect, we observed a small effect in the subcontrast for positive distractions indicating that right frontopolar processes were triggered during high compared to low workload. As we only observed this effect in the HbO subcontrast and not in the HbO main emotion contrasts related to negative or neutral distractions, it is possible that the distraction's impact and the subsequent requirement to suppress positive auditory stimuli through right-hemispheric (Grimshaw and Carmel, 2014) and frontopolar processes (Anticevic et al., 2010; Niendam et al., 2012; Mansouri et al., 2017) were relatively minimal.

### 4.3 Workload-independent negativity bias for neutral distractions

Since we did not observe right-hemispheric inhibitory processes triggered during positive distractions, our third hypothesis regarding differences between neutral and positive distractions during low workload was not confirmed. Regarding the comparison between neutral and negative distractions, we observed reduced right-hemispheric frontopolar activity, instead of the expected reduced left-hemispheric processing. Less cognitive control processes were triggered during neutral compared to negative distraction, independent of the workload level (HbO main emotion effect). These processes observed in the right frontopolar cortex do not entirely align with the asymmetric inhibition model (Grimshaw and Carmel, 2014).

We hypothesized a workload-dependent effect for neutral distractions, characterized by a negativity bias when experiencing high workload. However, one main finding of the study was that the negativity bias for neutral distractions was consistently present, irrespective of the workload level. Neutral and negative distractions elicited comparable activation patterns in the left OFC and IFG, compared to positive distractions, and exhibited no significant difference in left-hemispheric processing when contrasted against each other (HbO and HbR main emotion effects). The negativity bias for neutral distractions could be particularly pronounced for auditory stimuli, given their highly intrusive nature and the challenges associated with avoidance strategies (e.g., humans cannot close their ears and shifting the attentional focus away from auditory input is difficult; Berti and Schröger, 2001; Rau et al., 2020). Differences between the auditory and visual systems might also explain why other studies using visual emotional stimuli did not report a negativity bias for neutral distractions (García-Pacios et al., 2015a,b).

### 4.4 Left-hemispheric processing of emotional speech distractions

Since semantic processing and evaluation of emotional speech is associated with left-hemispheric activation in the IFG (Gabrieli et al., 1998; Zahn et al., 2000; Wildgruber et al., 2004; Friederici, 2012), we also hypothesized a left-hemispheric lateral processing bias for positive distractions during low workload. Interestingly, we observed lower HbR estimates and, thus, increased activity in the left IFG during positive distractions and low workload (HbR interaction effect). The subcontrasts between negative and positive distractions revealed a smaller effect in the low compared to the high workload contrast, indicating less difference between the subconditions in the left IFG recruitment. Consequently, the IFG involvement can be attributed to asymmetric inhibition processes on the one hand, but on the other hand, it may also be related to semantic processing and evaluation of the emotional speech (Gabrieli et al., 1998; Zahn et al., 2000), at least for positive distraction under low workload. Therefore, our findings indicate that participants could thoroughly process emotionally positive utterances during low workload and evaluate the stimuli regarding their self-related (social) relevance (Gabrieli et al., 1998; Zahn et al., 2000). We cannot rule out the possibility that sufficient cognitive resources were available to semantically process the negative and neutral emotional speech, hence engaging the left IFG besides its involvement in inhibitory processes. This is because we did not compare them to non-semantic negative and neutral utterances, but only to positive emotional speech. The prerequisite of available cognitive resources to process and evaluate the emotional speech is also supported by the HbO main workload effect, suggesting a workload-related modulation of left IFG recruitment. The left IFG was less activated during high workload scenarios, in which fewer cognitive resources were available and semantic processing was reduced. Thus, we conclude that the left IFG appears to play a crucial role not only in inhibiting negative distractions, but also in processing and evaluating emotional speech, when sufficient cognitive resources are available. Stimulus- and workload-specific effects are currently not integrated into the asymmetric inhibition model (Grimshaw and Carmel, 2014).

### 4.5 General discussion

We could pinpoint hemispheric asymmetries in the PFC involvement during the interaction between emotion and cognition in a close-to-naturalistic experimental setting. Consistent with previous studies and theories, our findings indicate the specific PFC areas, including the left IFG and OFC, are involved during interacting emotional and cognitive processes. Moreover, we extended the understanding of hemispheric-specific inhibitory processes during negative and positive, but also neutral distractions, by examining workload-dependent and speech-specific effects. The lateralized hemispheric processing in the PFC is influenced by both workload level and emotional speech distractions. Therefore, the availability of cognitive resources and specific stimulus characteristics of emotional distractions significantly influence hemispheric asymmetries during the processing and inhibition of the stimuli.

One main goal of the study was to investigate whether assumptions of the asymmetric inhibition model (Grimshaw and Carmel, 2014) hold true with naturalistic auditory emotional speech distractions and a human-machine monitoring task, under multiple workload levels. From our findings, we conclude that the proposed model fails to specify and address influencing factors such as increased workload or emotional language processing during the hemispheric asymmetric inhibition of emotional distractions. Furthermore, while the model suggests an interaction between emotional and cognitive processes, it does not specify the precise nature of this interaction. Through our study, we have contributed to a better understanding of how asymmetric inhibition processes in the PFC interact with different workload levels.

When investigating the interaction of workload and emotional speech distraction, we were confronted with the challenge of disentangling left hemispheric processes associated with inhibition from those related to semantic processing of emotional speech distractions. Future research could address this by incorporating non-semantic emotional stimuli, allowing for a better understanding of the interplay between workload and semantic processing and the allocation of left hemispheric activation between inhibition and speech-related processes.

Besides speech-related processes, various other factors can influence lateralized brain activation during different emotional distractions and workload levels. Previous research has shown that stimuli with emotional prosody tend to elicit stronger right-hemispheric processing compared to neutral ones (Buchanan et al., 2000; Lindell, 2006). In our study, we observed a slight decrease in right frontopolar activity during the inhibition of neutral compared to negative distractions. Hence, it is possible that emotional prosody played a role in modulating the asymmetric processing.

Our task required constant visuospatial processing, including considerations such as object distances from airport safety regions and frequent reallocation of attention. The reallocation occurred not only during performing the task (e.g., switching between the radar screen and control panel) but also during the inhibition of distractions to focus on goal-directed actions. These cognitive processes are positively correlated with task difficulty and workload (Chen and Spence, 2017), and, therefore, contribute to the observed brain patterns when comparing different workload levels. In addition, further experimental parameters like the number of visual stimuli and their timing may have affected hemispheric asymmetries in the processing (Stevens et al., 2011; Felisatti et al., 2020). Attentional and spatial processes tend to evoke right-lateralized brain activity, particularly in temporal, parietal, and occipital regions (Yamaguchi et al., 2000; Yovel et al., 2001; Han et al., 2002; Corbetta and Shulman, 2011; Thiebaut de Schotten et al., 2011; Chen and Spence, 2017; Bartolomeo and Seidel Malkinson, 2019). However, it is worth noting that certain frontal regions which are part of attentional networks, such as the vlPFC (Bartolomeo and Seidel Malkinson, 2019), may also have been influenced by these task-related components. Finally, although participants did not explicitly report specific strategies for solving the monitoring-control task, it is important to consider that various mnemonic strategies could have also modulated hemispheric processing (Dörfel et al., 2014; Picó-Pérez et al., 2017).

To investigate such influencing factors, researchers might expand fNIRS measurements to encompass the entire cortex. This could further provide an opportunity to examine communication processes between frontal and posterior regions (e.g., the temporal and parietal cortex) involved in the inhibition of emotional distractions and maintenance of task-related processes (Sörqvist et al., 2016; García-Pacios et al., 2017; Scheunemann et al., 2018). Another goal of future research could be the investigation of time signatures during subprocesses of the emotion-cognition interaction; for instance, by combining fNIRS with a high temporal resolution method like a magnetencephalography or EEG (García-Pacios et al., 2015a, 2017).

During the interpretation of our results, we encountered the issue that certain effects were observed in the HbR analysis but not in the HbO analysis (for general discussions, see Pinti et al., 2020; Kinder et al., 2022). Glotzbach et al. (2011) investigated the neuronal signatures of the prefrontal cortex (PFC) during emotion induction and regulation. Similar to our results, they also reported significant changes in HbR but not HbO concentration over the left PFC during the regulation of emotions. Although changes in the HbO concentration generally tend to be of higher amplitude and have a higher signal-to-noise-ratio (Pinti et al., 2020), some studies reported a caveat of potentially confounding physiological interferences (Kirilina et al., 2012; Haeussinger et al., 2014; Tachtsidis and Scholkmann, 2016). HbR concentration changes are suggested to be less influenced by systemic physiological artifacts like cardiac activity, respiration, or Mayer wave fluctuations (Obrig et al., 2000; Huppert et al., 2009b; Zhang et al., 2009; Pinti et al., 2020). Due to the small effects and inconsistencies between HbO and HbR, as well as our rather small sample size, we encourage other researchers and future studies to replicate the experiment in order to ensure the robustness of the observed effects of emotional speech distractions and workload levels.

Our choice of a close-to-naturalistic experimental paradigm is akin to a double-edged sword, offering advantages of increased ecological validity but also possessing inherent limitations due to less controlled settings. This approach carries a chance of unintended confounding factors, which can be difficult to disentangle, along with potential movement-related artifacts during the task. The introduced variability may mask small effects and result in reduced statistical power compared to highly controlled laboratory experiments. However, researchers have long advocated a two-pronged approach, consisting of complementary laboratory and naturalistic studies, as well as multi-method approaches (Parrott and Hertel, 1999). To answer the question of how emotional and cognitive processes interact and which factors decisively influence these processes, naturalistic studies are necessary to verify ideas and theories, developed and tested in the laboratory, under more realistic circumstances. The manipulation strength of the stimulus material and task plays a key role in close-to-naturalistic experiments (Peck et al., 2014; Westgarth et al., 2021). Although we chose a naturalistic database with semantically intelligible content for the emotional speech distractions (Burkhardt et al., 2005), sequences were still rather short and not embedded in a meaningful context. The lack of a meaningful context might have reduced the strength of emotional manipulation. Therefore, future studies could consider using validated emotional dialogues and longer sequences, providing a comprehensible and coherent context (Lingelbach et al., 2023).

### 4.6 Conclusion

To investigate PFC activation patterns of interacting cognitive and emotional processes, we performed a close-to-naturalistic fNIRS study with low and high workload task scenarios and concurrent emotional distractions differing in their valence. Our results revealed the following findings: (1) a cross-over interaction indicating workload-dependent hemispheric asymmetries during the processing of negative and positive distractions at different workload levels, (2) a workload-independent negativity bias for neutral speech distractions, and (3) potential additional left-hemispheric effects of emotional speech processing and evaluation during positive distractions with sufficient processing capacity. Through investigating workload-dependent and stimulus-specific effects, we have deepened the understanding of the factors that influence hemispheric asymmetries during the processing and inhibition of emotional distractions, as well as the interaction between emotion and cognition.

## Data availability statement

Raw data supporting this study are available from the project OSF repository at https://osf.io/49dqk/.

## Ethics statement

The studies involving humans were approved by the Ethics Committee at the Medical Faculty of the Eberhard-Karls-University and at the University Hospital Tübingen (ID: 827/2020BO1). The studies were conducted in accordance with the local legislation and institutional requirements. The participants provided their written informed consent to participate in this study.

## Author contributions

KL: Conceptualization, Data curation, Formal analysis, Funding acquisition, Investigation, Methodology, Project administration, Resources, Software, Supervision, Validation, Visualization, Writing – original draft, Writing – review & editing. SG: Data curation, Formal analysis, Software, Visualization, Writing – review & editing. MW: Conceptualization, Funding acquisition, Resources, Supervision, Writing – review & editing. MV: Resources, Writing – review & editing.
