# Supplementary material for: Workload-dependent hemispheric asymmetries during the emotion-cognition interaction: a close-to-naturalistic fNIRS study
Source: Front Neuroergon. 2023 Nov 15;4:1273810. doi: 10.3389/fnrgo.2023.1273810 (PMC10790862; doi:10.3389/fnrgo.2023.1273810)
Supplement: Supplementary file 1 [file Data_Sheet_1.PDF]

Supplementary Material

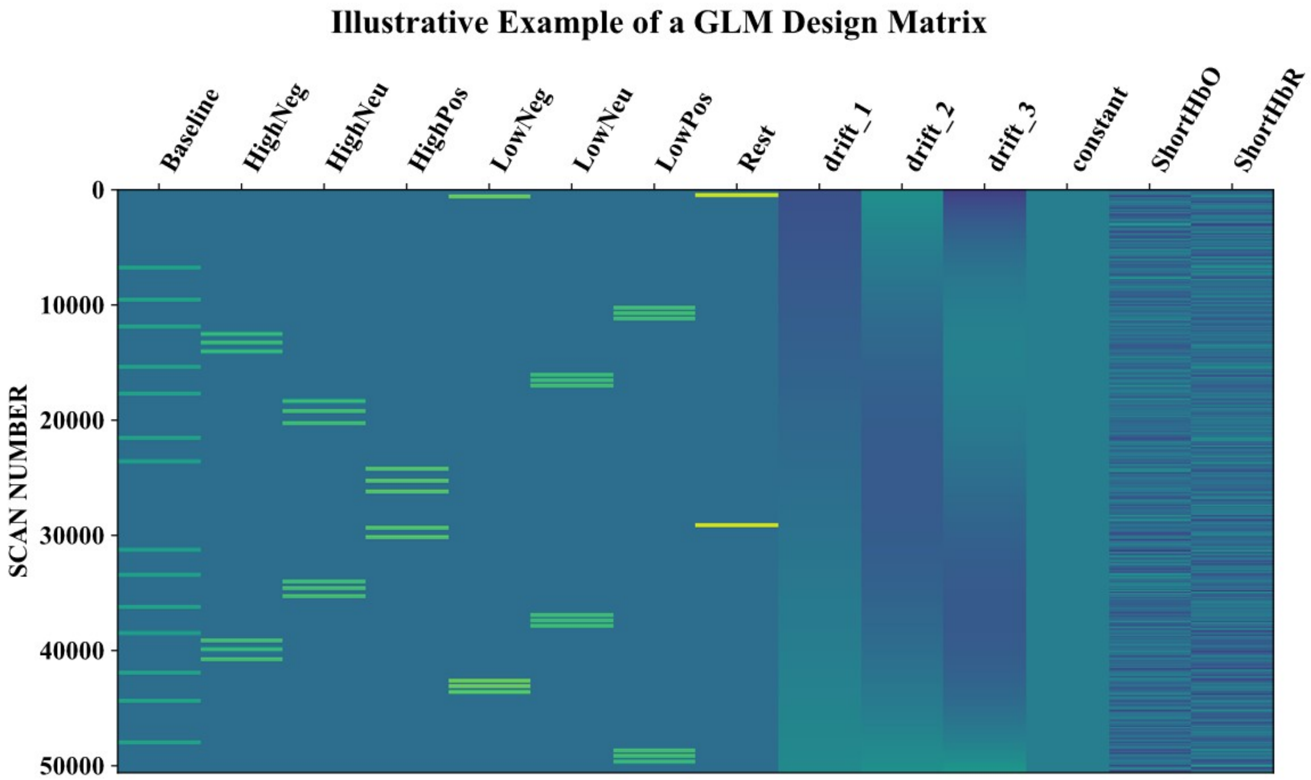

Figure S1. Illustrative example of the design matrix from one participant with the regressors of the first-level generalized linear model (GLM) represented as columns.

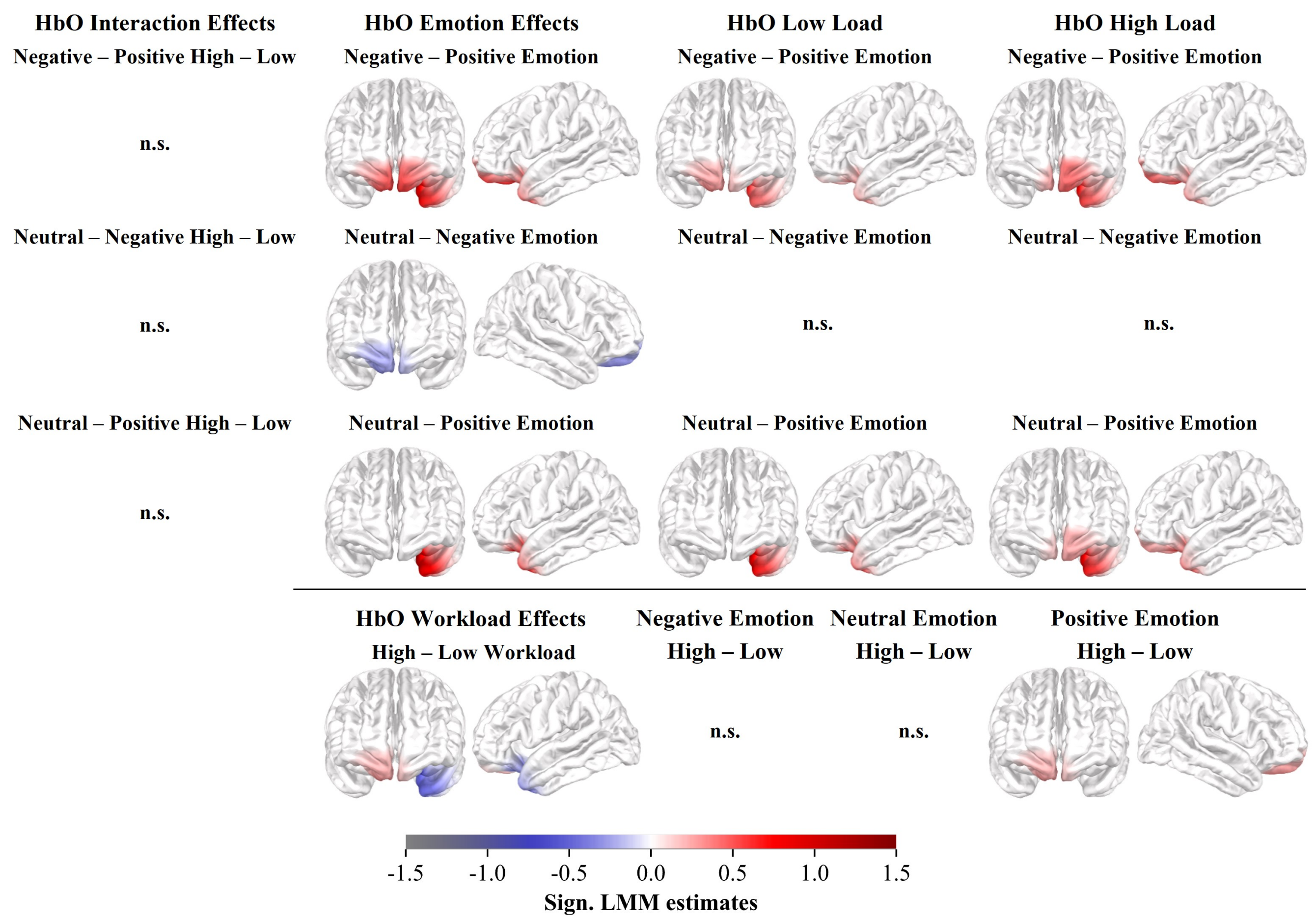

Figure S2. Overview of all interaction or main effects as well as subcontrasts of the involved subconditions with the significant second-level linear mixed-effects models (LMM) HbO estimates projected onto a 3-D brain surface.

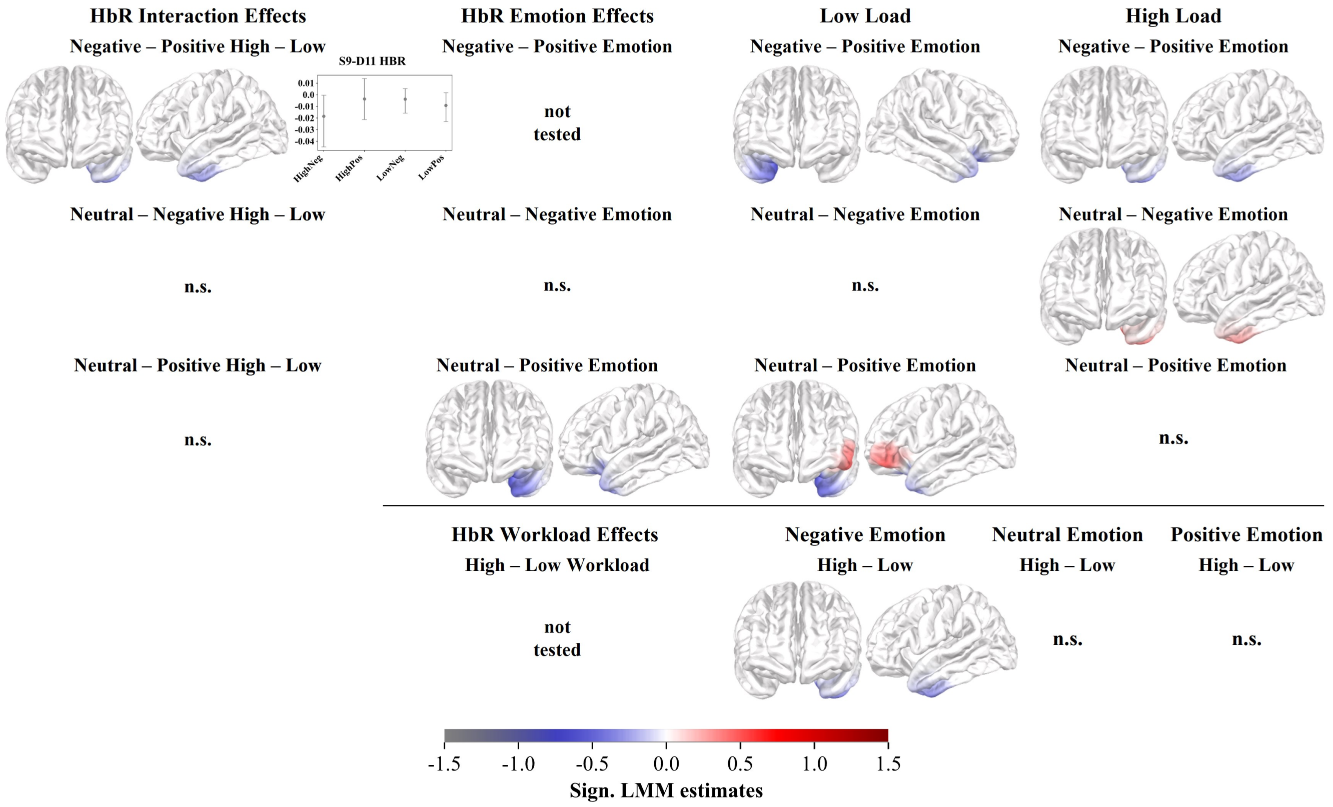

Figure S3. Overview of all interaction or main effects as well as subcontrasts of the involved subconditions with the significant second-level linear mixed-effects models (LMM) HbR estimates projected onto a 3-D brain surface.

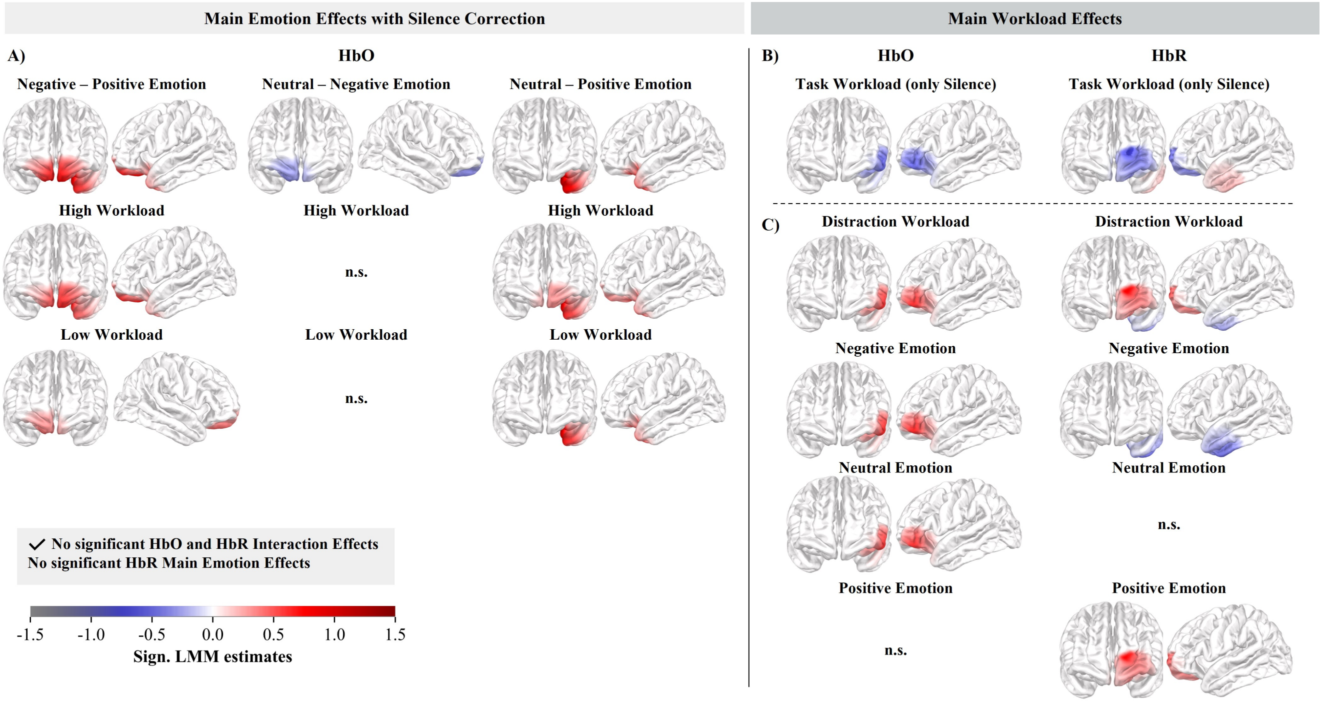

Figure S4. Overview of the significant effects of the second-level linear mixed-effects models (LMM) for the HbO and HbR analyses when subtracting the task-induced workload from each condition (Silence High and Silence Low). The additional analyses serve to investigate the combination of both factors - emotion and workload in the interactions. Further, they allow to investigate the brain activation patterns associated with workload induced solely by the task (B; Silence High - Silence Low) and the auditory distractions (C; ((Negative High - Silence High) + (Neutral High - Silence High) + (Positive High - Silence High)) - ((Negative Low - Silence Low) + (Neutral Low - Silence Low) + (Positive Low - Silence Low))).

1 ADDITIONAL ANALYSES USING THE SILENCE CONDITION

We included the silence condition to investigate the effects of the workload induced solely by the task, the workload induced by the distraction and the effect of emotion without task workload. The rationale was the following:

- By subtracting the silence conditions, the workload induced by the task should be mitigated. In the presence of a true interaction between the emotion and workload, we should not observe any significant interactions between these factors when subtracting the respective silence condition from each condition (e.g., Neutral High - Silence High; Negative High - Silence High, Positive High - Silence High, Neutral Low - Silence Low; Negative Low - Silence Low, Positive Low - Silence Low). Our results revealed no significant interaction effects after subtracting the silence condition (gray information box).
- Since subtracting the silence condition should primarily remove task-induced workload, the main emotion effects remain unaffected. The results of the main emotion contrasts should be comparable to those without subtracting the silence conditions. The results, where we subtracted the silence condition, demonstrated similar HbO main emotion effects compared to the original analyses (A). In the HbR analyses, we did not observe a significant main emotion effect when comparing neutral to positive

distractions, indicating that this particular contrast might have been partially influenced by task-induced effects (gray information box).

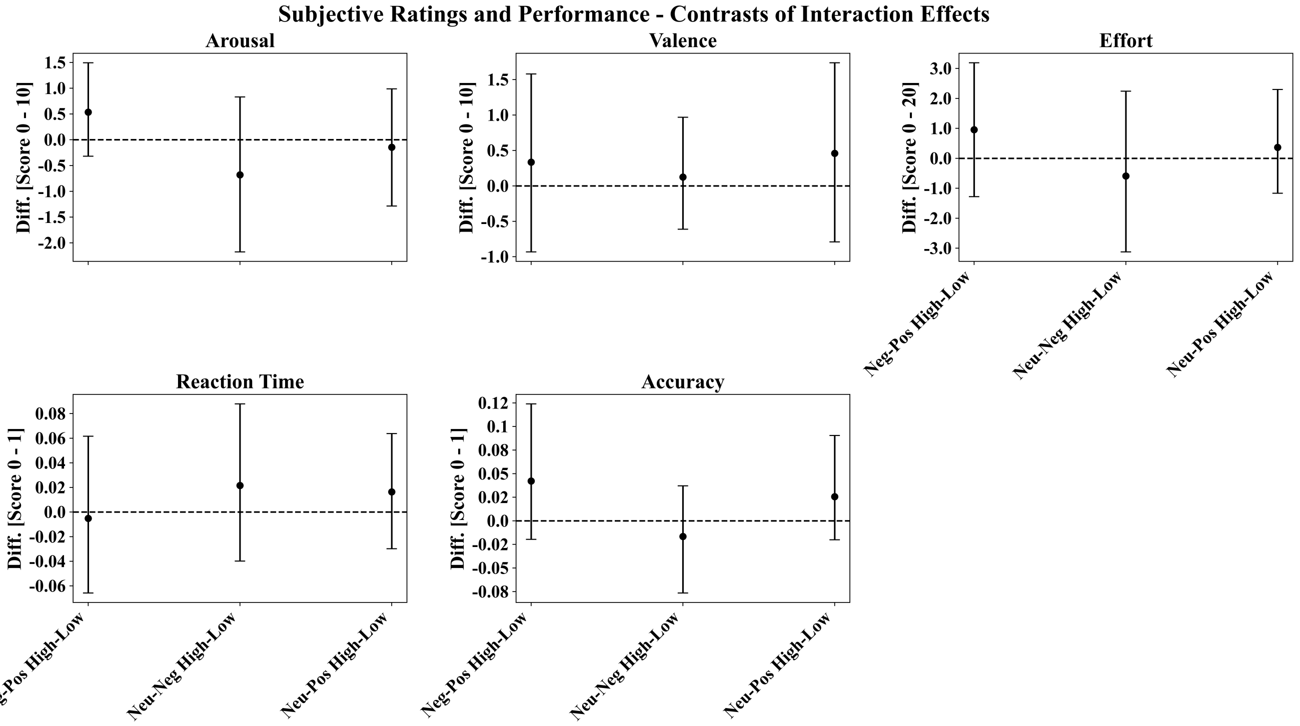

Figure S5. Interaction effects of the subjective ratings (arousal, valence, and effort) and performance measures (reaction time and accuracy). Dots and error bars illustrate the bootstrapped mean and its Bonferroni-corrected 95% confidence interval across participants per contrast.

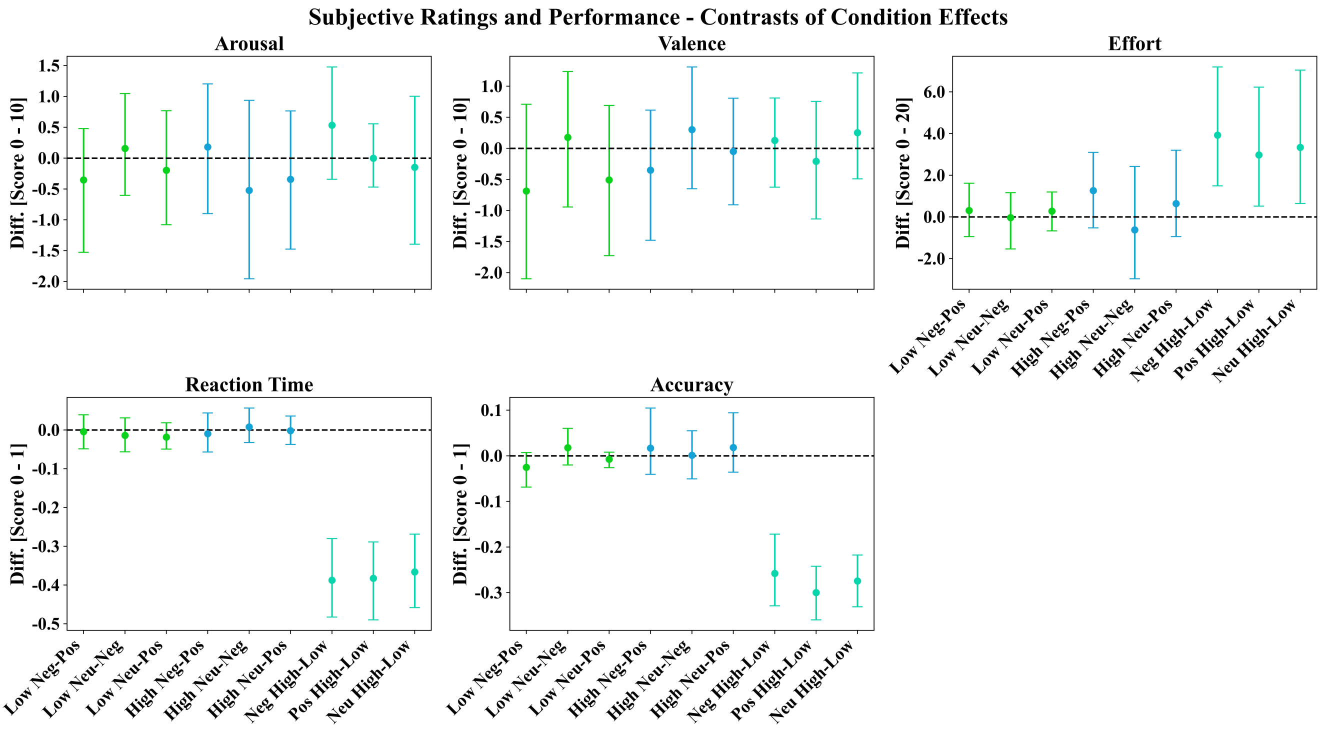

Figure S6. Subcontrasts between subconditions of the subjective ratings (arousal, valence, and effort) and performance measures (reaction time and accuracy). Dots and error bars illustrate the bootstrapped mean and its Bonferroni-corrected 95% confidence interval across participants per contrast.

Table S1. Overview of the channels, optodes, Brodmann Area (BA) correspondences (exceeding a specificity of above 20 %), and MNI coordinates based on the fOLD toolbox. L: left; R: right.

Channel	Source	Detector	Brodmann Area	X (mm)	Y (mm)	Z (mm)	Specificity (%)
1	S12 (FC2)	D3 (F2)	R BA8 - Frontal eye fields	24	26	55	58
			R BA9 - Dorsolateral prefrontal cortex				33
2	S12 (FC2)	D12 (FCz)	R BA6 - Pre-Motor and Supplementary Motor Cortex	14	13	66	63
			R BA8 - Frontal eye fields				35
3	S3 (Fz)	D3 (F2)	R BA9 - Dorsolateral prefrontal cortex	10	41	50	69
			R BA8 - Frontal eye fields				29
4	S5 (F4)	D3 (F2)	R BA9 - Dorsolateral prefrontal cortex	30	40	41	68
			R BA46 - Dorsolateral prefrontal cortex				22
5	S5 (F4)	D7 (FC4)	R BA9 - Dorsolateral prefrontal cortex	44	25	40	31
			R BA44 - pars opercularis, part of Broca's area				27
6	S12 (FC2)	D7 (FC4)	R BA6 - Pre-Motor and Supplementary Motor Cortex	39	12	54	38
			R BA9 - Dorsolateral prefrontal cortex				36
			R BA8 - Frontal eye fields				21
7	S2 (AF4)	D1 (AFz)	R BA10 - Frontopolar area	13	61	24	72
8	S2 (AF4)	D9 (Fp2)	R BA10 - Frontopolar area	25	63	9	69
			R BA11 - Orbitofrontal area				22
9	S6 (Fpz)	D9 (Fp2)	R BA10 - Frontopolar area	13	67	0	54
			R BA11 - Orbitofrontal area				45
10	S8 (AF8)	D9 (Fp2)	R BA10 - Frontopolar area	34	59	-2	31
			R BA11 - Orbitofrontal area				30
			R BA46 - Dorsolateral prefrontal cortex				20
11	S10 (F8)	D10 (F10)	R BA38 - Temporopolar area	52	30	-13	36
12	S10 (F8)	D14 (FT8)	R BA38 - Temporopolar area	57	21	-4	32
13	S14 (FC6)	D7 (FC4)	R BA44 - pars opercularis, part of Broca's area	56	12	33	41
			R BA6 - Pre-Motor and Supplementary Motor Cortex				40
14	S5 (F4)	D5 (F6)	R BA45 - pars triangularis Broca's area	46	38	24	71
			R BA46 - Dorsolateral prefrontal cortex				23
15	S10 (F8)	D5 (F6)	R BA45 - pars triangularis Broca's area	55	36	5	66
16	S14 (FC6)	D5 (F6)	R BA45 - pars triangularis Broca's area	58	24	18	53
			R BA44 - pars opercularis, part of Broca's area				29
17	S14 (FC6)	D14 (FT8)	R BA48 - Retrosubicular area	61	11	8	28
			R BA6 - Pre-Motor and Supplementary Motor Cortex				26
18	S8 (AF8)	D5 (F6)	R BA45 - pars triangularis Broca's area	48	46	5	44
			R BA46 - Dorsolateral prefrontal cortex				43
19	S2 (AF4)	D5 (F6)	R BA46 - Dorsolateral prefrontal cortex	40	50	16	47
			R BA45 - pars triangularis Broca's area				31
20	S3 (Fz)	D12 (FCz)	BA8 middle - Frontal eye fields	1	27	58	60
			BA6 middle - Pre-Motor and Supplementary Motor Cortex				24
21	S3 (Fz)	D1 (AFz)	BA9 middle - Dorsolateral prefrontal cortex	2	50	39	62
			BA10 middle - Frontopolar area				20
22	S6 (Fpz)	D1 (AFz)	BA10 middle - Frontopolar area	1	64	14	87
23	S11 (FC1)	D2 (F1)	L BA8 - Frontal eye fields	-23	26	56	64
			L BA9 - Dorsolateral prefrontal cortex				26
24	S3 (Fz)	D2 (F1)	L BA9 - Dorsolateral prefrontal cortex	-9	41	50	63
			L BA8 - Frontal eye fields				35
25	S4 (F3)	D2 (F1)	L BA9 - Dorsolateral prefrontal cortex	-31	39	41	67
			L BA46 - Dorsolateral prefrontal cortex				25
26	S4 (F3)	D6 (FC3)	L BA9 - Dorsolateral prefrontal cortex	-45	25	41	37
			L BA44 - pars opercularis, part of Broca's area				27
27	S11 (FC1)	D6 (FC3)	L BA6 - Pre-Motor and Supplementary Motor Cortex	-38	12	55	38
			L BA9 - Dorsolateral prefrontal cortex				35
			L BA8 - Frontal eye fields				23
28	S11 (FC1)	D12 (FCz)	L BA6 - Pre-Motor and Supplementary Motor Cortex	-13	12	67	73
			L BA8 - Frontal eye fields				26
29	S1 (AF3)	D1 (AFz)	L BA10 - Frontopolar area	-12	62	23	76
30	S1 (AF3)	D8 (Fp1)	L BA10 - Frontopolar area	-24	63	9	70
			L BA11 - Orbitofrontal area				20
31	S6 (Fpz)	D8 (Fp1)	L BA10 - Frontopolar area	-12	67	0	54
			L BA11 - Orbitofrontal area				45
32	S7 (AF7)	D8 (Fp1)	L BA11 - Orbitofrontal area	-33	59	-2	33
			L BA46 - Dorsolateral prefrontal cortex				25
			L BA10 - Frontopolar area				25
33	S9 (F7)	D11 (F9)	L BA38 - Temporopolar area	-49	31	-12	32
			L BA47 - Inferior prefrontal gyrus				32
34	S9 (F7)	D13 (FT7)	L BA38 - Temporopolar area	-54	21	-4	32
			L BA45 - pars triangularis Broca's area				24
35	S13 (FC5)	D6 (FC3)	L BA44 - pars opercularis, part of Broca's area	-55	12	34	48
			L BA6 - Pre-Motor and Supplementary Motor Cortex				36
36	S13 (FC5)	D4 (F5)	L BA45 - pars triangularis Broca's area	-56	24	20	53
			L BA44 - pars opercularis, part of Broca's area				34
37	S13 (FC5)	D13 (FT7)	L BA48 - Retrosubicular area	-59	11	9	32
			L BA6 - Pre-Motor and Supplementary Motor Cortex				24
38	S9 (F7)	D4 (F5)	L BA45 - pars triangularis Broca's area	-53	37	6	80
39	S4 (F3)	D4 (F5)	L BA45 - pars triangularis Broca's area	-46	39	26	73
			L BA46 - Dorsolateral prefrontal cortex				22
40	S7 (AF7)	D4 (F5)	L BA45 - pars triangularis Broca's area	-47	46	6	49
			L BA46 - Dorsolateral prefrontal cortex				43
41	S1 (AF3)	D4 (F5)	L BA46 - Dorsolateral prefrontal cortex	-39	50	17	49
			L BA45 - pars triangularis Broca's area				32

Table S2. Summary of the fixed effect factor Handedness when included in the second-level linear mixed-effects models (LMM).

Contrast	Estimate	Std. Error	*df*	*t*	*p*	*95% CI*
				**HBO**		
**Interaction**						
**Negative - Positive High - Low**	-0.308	0.129	16	-2.383	0.03	[-0.713, 0.1]
**Neutral - Negative High - Low**	0.481	0.178	16	2.705	0.016	[-0.04, 1.044]
**Neutral - Positive High - Low**	0.292	0.169	16	1.722	0.104	[-0.259, 0.898]
**Main Emotion**						
**Negative - Positive**	0.0	0.164	16	0.0	1.0	[-0.488, 0.55]
**Neutral - Negative**	0.008	0.132	16	0.058	0.954	[-0.435, 0.441]
**Neutral - Positive**	0.007	0.105	16	0.068	0.947	[-0.355, 0.336]
**Main Workload**						
**High - Low**	-0.253	0.138	16	-1.841	0.084	[-0.689, 0.199]
				**HBR**		
**Interaction**						
**Negative - Positive High - Low**	0.116	0.099	16	1.171	0.242	[-0.218, 0.408]
**Neutral - Negative High - Low**	-0.276	0.11	16	-2.505	0.023	[-0.632, 0.091]
**Neutral - Positive High - Low**	-0.186	0.099	16	-1.879	0.061	[-0.544, 0.14]
**Main Emotion**						
**Negative - Positive**	-0.086	0.11	16	-0.779	0.447	[-0.45, 0.294]
**Neutral - Negative**	0.078	0.123	16	0.639	0.532	[-0.334, 0.505]
**Neutral - Positive**	-0.008	0.098	16	-0.078	0.938	[-0.333, 0.306]
**Main Workload**						
**High - Low**	0.003	0.159	16	0.02	0.984	[-0.512, 0.503]

Table S3. Summary of the second-level linear mixed-effects model (LMM) for the HbR Interaction Effect Contrast Negative - Positive Emotion High - Low Load in the significant channel S9-D11.

	Estimate	Std. Error	*df*	*t*	*p*	*95% CI*
**Workload**	0.476	0.189	51	2.515	0.015*	[0.102, 0.843]
**Emotion**	0.479	0.189	51	2.536	0.014*	[0.105, 0.862]
**Workload * Emotion**	-0.645	0.267	51	-2.414	0.019*	[-1.179, -0.107]
